# Personality heterophily and friendship as drivers for successful cooperation

**DOI:** 10.1098/rspb.2023.2730

**Published:** 2024-03-27

**Authors:** Debottam Bhattacharjee, Sophie Waasdorp, Esmee Middelburg, Elisabeth H.M. Sterck, Jorg J. M. Massen

**Affiliations:** ^1^ Animal Behaviour and Cognition, Department of Biology, Utrecht University, Padualaan 8, 3584 CH Utrecht, The Netherlands; ^2^ Department of Infectious Diseases and Public Health, Jockey Club College of Veterinary Medicine and Life Sciences, City University of Hong Kong, Hong Kong SAR, People's Republic of China; ^3^ Centre for Animal Health and Welfare, Jockey Club College of Veterinary Medicine and Life Sciences, City University of Hong Kong, Hong Kong SAR, People's Republic of China; ^4^ Animal Science Department, Biomedical Primate Research Centre, 2280 GH Rijswijk, The Netherlands

**Keywords:** cooperative decision rules, partner choice, personality heterogeneity, friendship, primates

## Abstract

Cooperation is widespread and arguably a pivotal evolutionary force in maintaining animal societies. Yet, proximately, what underlying motivators drive individuals to cooperate remains relatively unclear. Since ‘free-riders’ can exploit the benefits by cheating, selecting the right partner is paramount. Such decision rules need not be based on complex calculations and can be driven by cognitively less-demanding mechanisms, like social relationships (e.g. kinship, non-kin friendships, dyadic tolerance), social status (e.g. dominance hierarchies) and personalities (social and non-social traits); however, holistic evidence related to those mechanisms is scarce. Using the classical ‘loose-string paradigm’, we tested cooperative tendencies of a hierarchical primate, the long-tailed macaque (*Macaca fascicularis*). We studied three groups (*n* = 21) in their social settings, allowing partner choice. We supplemented cooperation with observational and experimental data on social relationships, dominance hierarchies and personality. Friendship and dissimilarities in non-social ‘exploration’ and ‘activity–sociability’ personality traits predicted the likelihood of cooperative dyad formation. Furthermore, the magnitude of cooperative success was positively associated with friendship, low rank-distance and dissimilarity in the activity–sociability trait. Kinship did not affect cooperation. While some findings align with prior studies, the evidence of (non-social) personality heterophily promoting cooperation may deepen our understanding of the proximate mechanisms and, broadly, the evolution of cooperation.

## 1. Background

Cooperation, defined as the collaborative efforts of two or more individuals to achieve a common goal [1], is widespread in the animal social world and may exist in diverse forms, from breeding to hunting [2,3]. While humans are hyper-cooperative and exhibit incredibly complex dynamics of cooperation [4], several non-human animals also demonstrate surprisingly high intra- and interspecific cooperative tendencies [5]. Cooperation can serve as the building block for establishing and sustaining societies and may offer fitness benefits to group members [1,5,6]. Although the evolutionary mechanisms of cooperation are well studied from theoretical perspectives [7], what motivates individuals to cooperate with a partner remains poorly understood from a proximate mechanistic point of view. In other words, the question remains: How do individuals choose their cooperative partners? This is particularly important because cooperative interactions can present exploitable benefits. ‘Free-riders’ can reap the benefits without any contributions, thereby hampering the sustenance of cooperation [8]. While punishment rules can be enforced to stop exploitation [9,10], they may require considerable cognitive resources. Therefore, an efficient and preventive step would be to select reliable partners through ‘cognitively less demanding’ decision rules. Non-human animals may adopt various cooperative decision rules and employ suitable strategies to choose reliable partners. However, a comprehensive understanding of these mechanisms and their influence on partner choice remains limited due to the lack of holistic examination and the lack of partner choice in most experimental studies on cooperation.

Kin selection elucidates the basis for cooperative interactions among genetically related individuals [11]. Nevertheless, cooperation extends its reach beyond the confines of kinship and may endure through reciprocity among non-kin [12]. Members within a social group usually have ample opportunities for interactions, facilitating the occurrence of reciprocity. As a result, strategies such as ‘(generous) tit-for-tat’, ‘win-stay’ and ‘lose-shift’ may effectively operate within networks of repeated interactions to foster cooperation [13,14]. Conventionally, the cognitively sophisticated ‘calculated reciprocity’ was thought to drive cooperation [15], where cooperative investment is contingent on individuals' mental bookkeeping of the costs and benefits of interactions. A lack of empirical support for this reciprocity in animals due to several cognitive constraints (see [16]) led researchers to propose simpler mechanisms, like ‘attitudinal-’ [17] or ‘emotional reciprocity’ [18]. These forms of reciprocities suggest that individuals may develop emotionally mediated attitudes toward group members through social interactions [19–22], which help them make cooperative decisions. In addition, the relatively simple ‘symmetry-based reciprocity’ suggests that individuals can engage in reciprocity based on symmetrical variables like age, dominance rank, kinship etc. [15]. However, this hypothesis did not receive much support as reciprocity sustains even when the symmetrical variables are ruled out [21,23]. Although there is ambiguity regarding which of these mechanisms best predicts reciprocity [24], decision rules in selecting potentially cooperative partners based on low cognitive demands garnered the most support, explaining cooperative success in non-human animals.

Social tolerance, defined by an individual's willingness to allow others in proximity while obtaining a high-quality reward [25], has significant implications for cooperation. Enhanced social tolerance can drive prosocial and cooperative tendencies [4,26,27]. Therefore, varying dyadic tolerance levels within a group can determine the formation and maintenance of cooperative interactions. Like tolerance, friendship is also widely acknowledged as a catalyst for cooperation [28–30]. Friendship may form among individuals independent of genetic relatedness [31]. Social tolerance and friendships, however, can work independently despite having similar underpinnings. For instance, it is far-fetched to say that socially tolerant individuals are always friends, although friendship is typically driven by enhanced tolerance. The positive effect of friendship on cooperation success aligns with the idea that strong social bonds can enhance the fitness of the partners [32,33]. While this preferential selection may create a ‘biological market’, where friends are associated with assured benefits [34], choosing a friend is also more predictable and cognitively less demanding than choosing a stranger or non-friend while collaborating [35]. Thus, differentiated strengths of dyadic tolerance and friendships can be crucial in forming cooperative dyads and their maintenance and success in complex societies. Yet, a limited number of empirical studies investigating the direct influence of friendship on cooperation in non-human animals exist [36–38].

Another key proximate mechanism of cooperation is consistent inter-individual behavioural differences or personalities [39]. They are fundamental behavioural constructs that distinguish one individual from another and hold considerable evolutionary significance [40,41]. Empirical evidence suggests that people with prosocial personality traits, such as agreeableness, are more likely to cooperate [42]. Similarly, extraversion and cooperative success in humans are linked positively (see [43]). Interestingly, personality traits positively affecting cooperation, such as extraversion, sociability and agreeableness, are often grouped as ‘cooperative personality’ in humans [44]. To assess how personalities may influence cooperation, similarities (i.e. homophily) and differences (i.e. heterophily) in traits are often calculated at the dyadic levels. Several studies have concluded that similarity in personality, or personality homophily, is a strong predictor of friendship [45–48], which in turn may foster cooperation. A few studies on non-human primates so far show similar patterns: Barbary macaques (*Macaca sylvanus*) with similarities in the shyness-boldness personality axis cooperate with each other [49], and personality similarities are predominant in groups of cooperatively breeding species like common marmosets (*Callithrix jacchus*) [50,51]. Apart from primates, assortative mating based on personality similarity is linked to higher reproductive benefits in birds. For instance—a cross-fostering breeding experiment on zebra finches (*Taeniopygia guttata*) found that personality similarity within breeding pairs positively affects offspring fitness [52]. In cockatiels (*Nymphicus hollandicus*), mates with higher behavioural compatibility displayed better-coordinated incubation than those with lower behavioural compatibility [53].

In contrast to personality homophily, humans with dissimilar personalities can effectively engage in successful cooperative interactions, too. Cooperating individuals, each with a specific personality, may bring different problem-solving perspectives [54], thereby enhancing group performance [55,56]. Again, there have been reports about similar patterns in non-human animals, albeit scarcely. For example, rooks (*Corvus frugilegus*) successfully cooperate when dyads consist of a shy and a bold individual rather than two shy individuals [57]. In chimpanzees (*Pan troglodytes*), ‘impact’ males, dissimilar to the rest of the group, work as hunting catalysts [58]. Furthermore, disassortative mating strategies can be beneficial; they are often based on personality differences. Female rainbow kribs (*Pelvicachromis pulcher*) choose males of dissimilar levels of boldness [59], and great tits (*Parus major*) with varying explorative tendencies are more likely to be in a partnership [60]. While the prevailing hypothesis revolves around personality homophily as a predictor of cooperative success, the potential impact of personality dissimilarities on cooperation remains largely overlooked and consequently unexplored, especially in non-human animals. Nevertheless, it appears that while homophily in social personality traits (e.g. agreeableness, sociability, affiliation, cf. [61]) is predictive of cooperative success, heterophily in non-social traits (e.g. exploration, boldness, activity) may facilitate cooperation too. However, this remains a testable hypothesis to address whether and how individuals with personality (dis)similarity actively select one another as cooperative partners. Consequently, our understanding of how personalities influence cooperative decision-making and partner choice remains obscure.

Here we investigate the cooperative tendencies of a group-living primate species that has a matrilineal hierarchical social structure, the long-tailed macaque (*Macaca fascicularis*). While the existing covariation framework might suggest that a steep hierarchical structure could hinder cooperation [62], a growing number of empirical studies provide evidence of prosocial and cooperative tendencies in despotic societies [63–69]. Prosocial and cooperative behaviours seem to be selectively maintained through interdependence in these hierarchical systems [69]. Besides, rank similarities, i.e. low rank distances, can also be a strong mechanism favouring cooperation when hierarchies are relatively steep (see [70]). We employed a ‘loose-string paradigm’, wherein two individuals can attain rewards only by simultaneously pulling two loose ends of a single string [71]. Importantly, our study took place within the macaques' existing social groups, thereby allowing for free partner choice. While we did not explicitly assess partner choice by manipulation, any discernible pattern from our free partner choice set-up is expected to indicate the underlying mechanisms efficiently. In addition to cooperation, we experimentally measured the long-tailed macaques’ dyadic tolerance levels. Finally, we supplemented cooperation and tolerance with data on friendships, dominance hierarchy and personalities, which were collected using extensive behavioural observations and experimental assays.

We hypothesize that individuals will use cooperative decision rules based on kinship, social tolerance, friendships (marked by contact sit, including both kin- and non-kin-based friendships) and dominance rank relationships. We predict that the likelihood of cooperation and its success would be positively associated with kinship, social tolerance and friendship, whereas a negative effect of disparity in dominance ranks is expected. Finally, we hypothesize that personality will play a significant role in shaping both the establishment and success of cooperative dyads. Nonetheless, due to complex personality trait characteristics, we expect personality homophily and heterophily to influence cooperation at the trait level independently. In particular, we predict that homophily in affiliation and sociability-like traits (i.e. social personality traits) and heterophily in exploration and activity-like (i.e. non-social personality traits) traits, as potential mechanisms governing partner choice, will be positively linked to cooperation and its success.

## 2. Methods

### (a) Subjects and housing

We studied three social groups of captive long-tailed macaques residing at the Biomedical Primate Research Centre (BPRC) in Rijswijk, the Netherlands. The first group (Gr.1) consisted of 15 individuals, out of which four were under the age of 1 year at the beginning of the study and thus not included (*n* = 11). Among the remaining 11 individuals were seven adult females and one adult male, all above 3 years of age, as well as two juvenile females and one juvenile male between 1 and 3 years old. The second group (Gr.2) originally had 18 individuals but was reduced to 17 as one male was removed early during the study due to compatibility issues with conspecifics (*n* = 17). The remaining 17 individuals comprised 10 adult females, one adult male, two juvenile females and four juvenile males. The third group (Gr.3) consisted of four adult males (*n* = 4). The study was conducted in the macaques' existing social groups and home enclosures. We neither separated the individuals from their social groups nor made forced dyads. The demographic description of the individuals is provided in electronic supplementary material, table S1. See additional methods, electronic supplementary material, for husbandry protocol.

### (b) Experimental design and data collection

#### (i) Loose-string paradigm

We conducted a ‘loose-string paradigm’ to assess cooperation in this study [71–74]. The experimental apparatus consisted of a sliding platform (width = 1.1 m) on top of a larger wooden base (width = 1.3 m). The sliding platform had two feeding trays made of plastic positioned on each end of the section that faced the enclosures. The placement was done in such a way that prevented one individual from reaching both feeding trays simultaneously. The opposite section had a hard plastic handle attached, which the experimenter could pull to move the platform. On the side of the handle, two metal loops were anchored. Depending on the experimental phase, the string(s) were either attached to or inserted through the metal loops (see below). Throughout the experiment, corn, sunflower seeds or peas were used as food rewards alternately, as advised by the in-house veterinarians. The macaques were highly motivated to receive each item as a reward (D Bhattacharjee, personal observation) and did not show any preference (when the cooperation test trial was successful) for one over others.

In Gr.1, the experiment commenced between November 2021 and early February 2022. We tested Gr.2 between July and October 2021. Gr. 3 was tested between June and August 2021. Experiments were conducted outdoors for Gr.2 and Gr.3, whereas, due to low temperatures during the winter months, the experimental set-up was placed indoors for Gr.1. We tested the macaques one to 3 days a week in their existing social group settings, and experiments lasted a maximum of 3 h per day. The cooperation experiment consisted of three different phases (cf. [72,73])—(i) habituation, (ii) training and social tolerance and (iii) testing. The participating individuals were identified for all trials during those phases. We provide a brief summary of the cooperation experiment in the following paragraph. See additional methods, electronic supplementary material, for details on the experimental protocol and the criteria used for the different phases.

Individuals in the habituation phase were allowed to familiarize themselves with the apparatus and eliminate any potential bias of neophobia voluntarily. We placed food rewards on the feeding trays of the apparatus so that individuals could interact with the apparatus (and obtain rewards). In the training and social tolerance phase, individuals could self-train themselves with the pulling mechanism of the apparatus, based on which we also measured the tolerance of the macaques to their group members. We conducted a total of 18 sessions per group, with each session having 20 trials. Finally, we tested the cooperative behaviour of the macaques in the testing phase. Individuals had to simultaneously pull the two loose ends of a string to move the platform and access the food rewards (electronic supplementary material, movie S1). If one individual pulled, the string became unthreaded, and the platform did not move from its initial position. A total of 30 sessions were conducted per group, each with 20 trials. D.B. tested Gr.1, and S.W. performed the tests at Gr.2 and Gr.3. The macaques were familiar to both experimenters. We recorded the study using Canon Legria HF G25 and Sony FDR AX100E 4k video cameras mounted on tripods. Moreover, for Gr.2 and Gr.3, an instant coding sheet was maintained and later compared with the videos.

#### (ii) Personality

Individuals' personality scores were the same as reported in a previous study [75]. Here, we briefly summarize the methods and findings for convenience. We took a multi-method approach of behavioural observation and experiments to assess long-tailed macaques' personality traits (cf. [76]). A ‘bottom-up’ approach was used to avoid any subjective bias of predetermined clustering of behavioural variables. All 32 individuals tested in the current study were observed in two phases (with an interval of at least two months; see [75] for dates of data collection) using 20 min-long continuous focal follows (Phase 1: mean ± s.d. = 159.76 ± 2.67 observation minutes (min) per individual; Phase 2 = 159.89 ± 2.64 min per individual) in their existing social group settings. In addition, novelty experiments were conducted during the same phases to capture rare behaviours, like task solving, persistence and exploration. Food puzzles (pipe and wooden maze), novel food items (dragon fruit and rambutan/starfruit) and novel objects (egg container and massage roller) were repeatedly used, again with intervals of at least two months of in between. As personality traits are considered inter-individual differences that are consistent over time [39], the repeatability of all 74 variables (see [76] for ethogram) was checked between the two phases using an intraclass correlation analysis (ICC(3,1)). Thirty-three repeatable variables were obtained with ICC values ≥ 0.5; after investigating the communality scores, 12 of them (communality score range: from 72.28% to 94.14%) were finally included in a principal component analysis, which provided us with three non-correlating dimensions or personality traits, explaining 78% of the observed variance. We named the three dimensions ‘activity–sociability’, ‘Affiliation’ and ‘Exploration’. activity–sociability consisted of the following variables—*approach*, *leave*, *follow*, *pass by*, *leave passive*, *sit* and *social play*; except for *sit*, all other variables were loaded positively. Affiliation had two positively loaded variables: *proximity* and *groom*. Finally, the personality trait exploration included positively loaded *object manipulation*, *handling container* and *hang* variables. Affiliation and exploration implied social- and non-social traits, respectively. It is worth noting that we labelled the trait activity–sociability to incorporate activity- and sociability-related variables. With the exception of *social play*, all other variables could account for the activity component without necessitating direct social interactions. Consequently, this particular trait, even though labelled as activity–sociability, can be attributed to the general activity of the individuals to a large extent. Individual personality scores from each trait were extracted.

#### (iii) Dominance hierarchy and friendship

Dominance hierarchies were known and reported in [75] for all three groups. A Bayesian Elo-rating method [77] was used to construct the hierarchies based on unprovoked submissive behaviours—*avoid, be displaced, silent bared-teeth, flee* and *social presence*, recorded during the focal observations described above. These variables were independent of the variables used for personality assessment. Individuals were placed according to their ordinal ranks in the hierarchy for the three groups separately.

Empirical evidence suggests that sitting in body contact is a reliable indicator of relationship quality or friendship [48,78]. Therefore, we used *contact sit* as a proxy for friendships, defined by two individuals sitting or lying together with body parts other than limbs physically touching. Due to their use in personality assessment, we could not use other related metrics, like grooming, to combine with contact sit for a composite measure of friendship. Thus, the durational variable of contact sit was independent of the personality trait-related variables, making it particularly useful for the analyses in this study. From continuous focal observations (see [75]), the duration of contact sit was extracted for all within-group dyads.

### (c) Data preparation

#### (i) Cooperation

Cooperation (also synonymously used with cooperative success) was defined as when two individuals in the testing phase pulled the two loose ends of the string and obtained rewards without monopolization. It was coded as a binary variable (yes/no). Furthermore, we counted the number of times individuals in a particular dyad cooperated. Two experimenters (D.B. and S.W.) coded the cooperation data. Based on 20% of the data, inter-rater reliability was calculated and found to be excellent (Cohen's kappa = 0.97). Another person, unaware of the hypotheses and design of the study, coded 20% of the data as well; the reliability was found to be excellent (Cohen's kappa = 0.94).

As mentioned, after the cooperation studies, a 3-year-old male had to be separated from Gr.2 and thus was not assessed for personalities. We did not include the concerned individual in the cooperation data analyses. Consequently, all trials were removed where this individual was present. Unlike the 600 trials in the testing phase of the other two groups, we obtained only 459 trials from Gr.2. Similarly, the number of social tolerance trials differed between Gr.2 (192 trials) and the other two groups (each 360 trials). In Gr.1, Gr.2 and Gr.3, a total of seven (participation rate = 63.6%), 10 (59%) and 4 (100%) individuals participated in the testing phase (i.e. in at least in one trial), respectively. All 21 individuals successfully completed the habituation, training and social tolerance phases (see electronic supplementary material, table S1). Theoretically, each participating individual had the opportunity to form a dyad with the other participating group members. In line with this, all potential combinations of dyads were constructed separately for the three groups (potential number of dyads: Gr.1 = 21, of which 4 were kin dyads; Gr.2 = 45, of which 21 were kin dyads; and Gr.3 = 6, no kin dyads were present), and data on the probability or likelihood and magnitude of cooperation were used for further analyses. Due to the zero-inflated nature of the data, a hurdle approach was considered to analyse cooperation [79], where we first looked at the probability or likelihood (i.e. did a dyad cooperate at least once or not?) and then the magnitude of cooperation (i.e. number of successful trials when a dyad cooperated at least once; success > 0).

#### (ii) Social tolerance

We used data from the social tolerance test phase to measure dyadic tolerance levels. Since we conducted the experiment in social group settings and individuals can have varying tolerance levels to their group members, our method considered all opportunities when two specific individuals could co-feed or monopolize. We counted the numbers of times two specific participating individuals obtained food in the presence of each other without any monopolization (i.e. ‘co-feeding’ events). This was divided by the sum of those two individuals obtaining food alone (or monopolization). The resulting values were normalized for each group and used as social tolerance scores. The tolerance score ranged from −1.08 to 6.05 (median = −0.21), with higher values indicating higher tolerance within a dyad.

#### (iii) Personality differences

The absolute differences in personality scores were calculated separately for each dyad for the three traits. activity–sociability, affiliation and exploration had ranges of 0.03–3.42 (median = 1.13), 0.02–4.86 (median = 1.01) and 0.008–3.05 (median = 1.13), respectively, with higher values suggesting greater dyadic personality differences.

#### (iv) Friendship

We extracted the observed duration of contact sit for all within-group dyads. The duration of contact sit per dyad was divided by the total observation time of a dyad to obtain the rate. Dyadic contact sit rates were further divided by the mean rate of a group [32,80]. Finally, the resulting values were *z*-transformed at the group level to get contact sit scores. A score range of −0.52 to 2.85 (median = −0.42) was obtained, with high scores indicating prolonged sitting in body contact, i.e. stronger friendships between dyad members than the average dyad in their corresponding group.

### (d) Statistics

All statistical analyses were conducted in R (v. 4.3.0) [81]. We first compared the number of trials in the testing phase where an individual pulled the rope in the presence versus absence of participating partners using a Wilcoxon signed-rank test (paired-sample). Although delayed control trials can explain individuals' understanding of the need for partners [71], our measure can act as a potential indicator for the same, i.e. the requirement of a partner to obtain rewards successfully. The proportion of successful cooperation across sessions was checked for the three groups using Spearman rank correlations. As mentioned before, our measure of friendship was based on a single metric of dyadic contact sit since we could not use other related metrics like grooming due to its use in the personality assessment (cf. [75]). We did, however, investigate the convergent validity, i.e. we examined correlations between dyadic contact sit and grooming at the group level, using Spearman correlation tests. We found strong positive correlations between the two, suggesting that dyadic contact sit sufficiently captured friendships (see electronic supplementary material, additional methods, for details). Since personality homophily can predict friendships, we first checked whether there were significant correlations between contact sit scores and differences in each personality trait using Spearman rank correlations. Similarly, we performed a correlation test between contact sit scores and dyadic social tolerance.

Using a hurdle approach, generalized linear mixed-effect model (GLMM) analyses were performed to investigate the likelihood and magnitude of cooperation (see electronic supplementary material, additional methods, for more details). The first model (for likelihood) had cooperation success as the response variable (yes/no) with a binomial error distribution. By contrast, the other model had the number of such successful cooperation trials (i.e. magnitude, success > 0) as the response variable and used a Poisson error distribution. Additionally, this model included the number of trials in the testing phase as an offset term to account for the variations. Both models included the following fixed effects—dyadic personality difference scores (the three traits were included separately), dyadic social tolerance scores, friendship scores (i.e. dyadic contact sit scores), age of individuals, sex composition (female–female/female–male/male–male), age difference (in years), dominance rank distance and kinship (yes/no; mother–offspring, siblings), and random effects—identities of the two individuals and identities of the dyads nested within groups. Age, rank distance and age difference were *z*-transformed to improve model fit and avoid convergence issues. Null models for both likelihood and magnitude consisted of their corresponding response variables, the control variable (individual age) and the random effects.

GLMM analyses were conducted using the *glmmTMB* package [82]. Collinearity among the fixed effects was checked using the *performance* package [83] and a variation inflation factor (VIF) value of less than 5 was considered as low or no collinearity [84]. Null versus Full model comparisons were checked with the help of ‘lrtest’ function of the *lmtest* package [85]. Model selection was done by comparing the *Akaike Information Criterion* values (with ΔAIC ≥ 2 as the threshold) of the models where fixed effects had acceptable VIFs and residuals followed all normality assumptions. Model diagnostics (residual normality, dispersion and outliers) were investigated using the *DHARMa* package [86]. The significance value (*α*) was set at 0.05. Only best-fitted models are reported in the results.

## 3. Results

The percentages of successful cooperation were 45.5%, 44.6% and 32.16% for Gr.1, Gr.2 and Gr.3, respectively. Out of the 72 potential dyads from all three groups, at least one successful cooperation trial was found in 27 dyads (where 11 dyads were kin-based). Notably, the 21 individuals (see §2) cooperated successfully in a total of 671 testing trials (median value = 10 successful trials per dyad) spread among those 27 dyads (see electronic supplementary material, table S2 for details). In Gr.1, 85% of cooperating dyads had a consistent individual, whereas in Gr.2, 50% had a consistent individual. Unlike Gr.1 and Gr.2, all potential dyads cooperated in Gr.3. The two mixed-sex groups (Gr.1 and Gr.2) exhibited cooperation at a comparable level (Fisher's exact test: *p* = 0.80) but higher than the smaller all-male (Gr.3) group (Gr.1 and Gr.3: *p* < 0.001; Gr.2 and Gr.3: *p* < 0.001). Overall, individuals pulled the string significantly more often with a partner than when alone (Wilcoxon signed-rank test: *z* = 2.279, *p* = 0.02), positively indicating that they seem to have at least some understanding of the need for a partner to obtain rewards. For all three groups, a positive trend or a significant correlation was found between the proportion of successful cooperation and test sessions (Spearman correlation test, Gr.1: *ρ* = 0.30, *p* = 0.10; Gr.2: *ρ* = 0.34, *p* = 0.06, Gr.3: *ρ* = 0.79, *p* < 0.001), indicating an effect of learning.

We found no evidence of a correlation between friendship and personality differences (activity–sociability: rho = −0.20, *p* = 0.09; affiliation: rho = −0.09, *p* = 0.44; exploration: rho = −0.06, *p* = 0.59). Similarly, no correlation was found between friendship and dyadic social tolerance (rho = 0.002, *p* = 0.98). Therefore, instead of covariates, individual effects of personality differences, friendship and tolerance were tested in the subsequent statistical models. Nonetheless, we checked for collinearity among these variables for further confirmation. Additionally, no difference in friendship between kin and non-kin dyads was found (Mann–Whitney *U* test: *z* = 1.355, *n*_kin_ = 25, *n*_non-kin_ = 47, *p* = 0.175).

**Figure 1.**
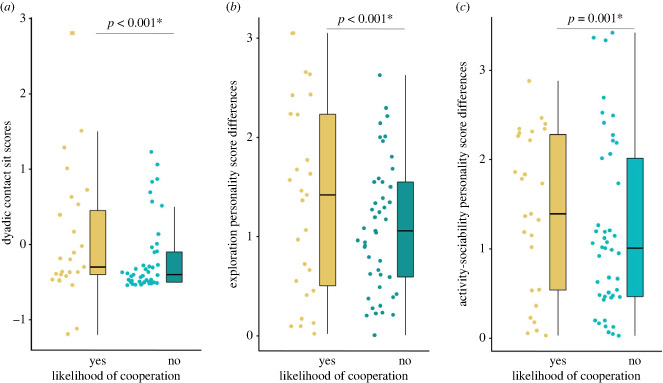
Effects of contact sit, exploration and activity–sociability differences on the likelihood of cooperation. (*a*) A box-whisker plot showing the contact sit scores for cooperative and non-cooperative dyads (GLMM: *p* < 0.001); (*b*) a box-whisker plot showing the personality trait exploration score differences for cooperative and non-cooperative dyads (GLMM: *p* < 0.001); (*c*) a box-whisker plot showing the personality trait activity–sociability score differences for cooperative and non-cooperative dyads (GLMM: *p* = 0.001). Boxes represent interquartile ranges and whiskers represent the upper and lower limits of the data. The horizontal bars within the boxes represent the median values. Outlier highlighted by square.

Based on the best-fitted model (see electronic supplementary material, tables S3a–S3c, for model selection), friendship, activity–sociability and exploration trait differences predicted the likelihood of cooperation success, i.e. whether a pair cooperated successfully at all (electronic supplementary material, table S3c). A positive association was found between the likelihood of cooperation success and friendship (GLMM: *z* value = 3.851, *p* < 0.001, figure 1*a*). The higher differences in exploration trait scores predicted the likelihood of cooperation (GLMM: *z* value = 3.518, *p* < 0.001, figure 1*b*). Similarly, dyads with larger differences in activity–sociability trait scores were more likely to cooperate than dyads with smaller differences (GLMM: *z* value = 3.227, *p* = 0.001, figure 1*c*). The full model differed significantly from the null model (likelihood ratio test: *χ*^2^ = 34.65, *p* < 0.001). We found no collinearity among the fixed effects (VIF range = 1.01–4.29).

**Figure 2.**
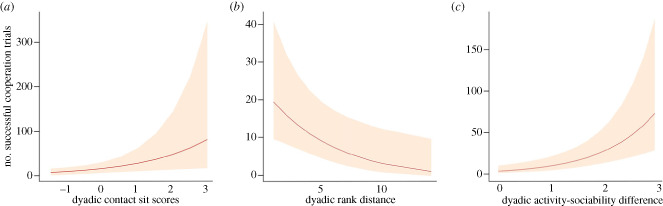
Effects of contact sit, rank distance and activity–sociability differences on successful cooperation. (*a*) GLMM effect plot showing the predicted effect of dyadic contact sit on the number of successful cooperation trials (*p* < 0.001); (*b*) GLMM effect plot showing the predicted effect of dyadic rank distance on the number of successful cooperation trials (*p* < 0.001), (*c*) GLMM effect plot showing the predicted effect of activity–sociability personality difference on the number of successful cooperation trials (*p* = 0.001). Shaded areas in the plots indicate 95% confidence intervals.

While looking at the magnitude of cooperation (for model selection, see electronic supplementary material, tables S4a–S4c) of those dyads that cooperated at least once (second hurdle of the hurdle model), we found that friendship, dominance rank distance and activity–sociability trait differences significantly predicted the magnitude of cooperation (electronic supplementary material, table S4c). A higher cooperation success was associated with friendship (GLMM: *z* value = 2.619, *p* = 0.008, figure 2*a*) and lower dominance rank distance (GLMM: *z* value = −2.548, *p* = 0.01, figure 2*b*). Furthermore, a larger difference in activity–sociability trait scores was linked to higher cooperation success (GLMM: *z* value = 4.582, *p* < 0.001, figure 2*c*). We did not find any effects of affiliation and exploration score differences and social tolerance on the magnitude of cooperation (electronic supplementary material, table S4c). The full model differed significantly from the null model (likelihood ratio test: *χ*^2^ = 31.95, *p* < 0.001). No collinearity among fixed effects was found (VIF range = 1.01–1.73).

## 4. Discussion

Using a free partner choice experimental set-up, we investigated the effects of social relationships (kinship, non-kin friendships, dyadic tolerance), social status (dominance hierarchies) and personalities as proximate mechanisms of cooperation in a hierarchical group-living primate. Larger mixed-sex groups exhibited higher cooperation than a relatively smaller all-male group. Friendship predicted the formation of cooperative dyads and their success. Incongruence in dominance position had an inverse association with cooperative task success, suggesting that individuals with similar ranks in the hierarchy were more efficiently involved in cooperation. To our surprise, kinship and social tolerance did not influence cooperation. While we found no support for homophily in social personality traits fostering cooperation, heterophily in the non-social personality traits was linked to the formation of cooperative dyads and their success. This study provides valuable insights into the proximate mechanisms underlying partner choice in cooperation, allowing us to better comprehend the evolution of cooperative interactions.

Group-level variations in altruistic (i.e. prosocial and cooperative) tendencies are often ignored. Even though species-typical patterns are of utmost importance for understanding the evolutionary trajectories of cooperation, socio-ecological and ‘group-specific’ factors can contribute to considerable inter-group differences within a species (see [65]). The social compositions of our study groups, albeit in relatively smaller group sizes, represented long-tailed macaque social organizations that are typically found in the wild [87,88]; mixed-sex groups that include philopatric females from different matrilines represent a more stable form of social organization than the bachelor male (i.e. dispersing sex) groups. This stability in social organization might have resulted in higher cooperation in mixed-sex than in all-male groups. Nonetheless, even within all-male groups, strong social bonds may form (e.g. political coalitions), which may or may not be facilitated by kinship [31,89]. Interestingly, however, unlike in the all-male group, cooperation success in mixed-sex groups depended heavily on dyads formed between a few specific individuals. Additionally, some individuals were consistent in most or at least half of the dyads that successfully cooperated. This could suggest monopolization of the apparatus or high selectivity in partners with whom cooperation can be initiated and sustained in despotic societies. Following a high degree of interdependence among individuals in despotic societies (see [69]), selectivity in prosocial and cooperative behaviour is a more plausible explanation than monopolization. However, it is worth mentioning that 21 individuals participated and of the 72 potential dyads, 27 unique dyads successfully cooperated at least once, making the likelihood and magnitude analyses viable. It would be valuable to conduct similar cooperation studies in relatively egalitarian macaque societies to see whether such a pattern prevails, or test despotic societies with access to multiple cooperation apparatuses.

In general, hierarchical matrilineal societies exhibit kin bias, and thus, genetically related individuals may engage in nepotistic cooperation [90]. However, we did not find any evidence of nepotistic cooperation in this study despite the participation of both kin and non-kin dyads. A previous study on long-tailed macaques found a positive association between kinship and prosocial tendencies [63]. Interestingly, when prosocial choices became costly, such associations seemed to disappear [67]. Constraints on nepotism, like the immediate exertion of competition within matrilines and the subsequent need for non-kin allies, can explain the lack of evidence of kinship influencing cooperation. In Japanese macaques (*Macaca fuscata*), non-kin alliances are formed and become more advantageous than kin-based allies, comprised of matriline members [91]. Similarly, the majority of the highly cooperative dyads are unrelated or distantly related in chimpanzees [92]. Finally, in a food-sharing experiment, long-tailed macaques do not discriminate between kin and non-kin members [93]. Hence, growing empirical evidence shows higher cooperative success among non-kin than kin members. But why non-kin dyads are more effective than kin dyads, and particularly in what settings, remains to be investigated. Nonetheless, taken together, kinship might not be the best predictor of cooperation in hierarchical primates; instead, its simpler underlying mechanism, like familiarity, may be at play when choosing a partner.

Incongruence in dominance rank can be detrimental to cooperation [94,95] and our study found support for this effect. The intensity of cooperation increased with decreasing rank distance. Non-human primates are known to assess their own and other group members' social ranks [96]. Besides, in despotic societies, a relatively steep and linear dominance hierarchy provides clear predictability of the directions of agonistic interactions [97]. Therefore, cooperating more with individuals from similar dominance ranks can be a sustainable strategy to minimize exploitation and avoid potential conflicts (see [72]). However, the likelihood of cooperation did not depend on rank distance, indicating an influence of other proximate mechanisms triggering cooperative dyad formation. The non-significant and significant effects of rank distance on the likelihood and magnitude of cooperation, respectively, suggest that repetitive interactions were necessary for dominance hierarchy to be used as a decision rule for partner choice.

Our findings align with previous studies indicating that friendship paves the path for cooperation [30,78,98]. Individuals were more likely to choose friends over non-friends to build cooperative dyads and subsequently became highly successful. However, a previous study on captive long-tailed macaques found no effect of social bonds on cooperative success [68]. Such a contrasting finding could be attributed to methodological differences and the use of forced dyads. Also, unlike contact sit in the current investigation, the previous study used proximity (and grooming instances) to measure social bond strengths. This potentially indicates that sitting in body contact is a powerful proxy for friendship in non-human primates, especially in hierarchical societies, where grooming can be directed towards dominant individuals [48], but needs further investigation. We found that friendships were independent of kin relationships. Thus, choosing a group member as a cooperative partner seems to be driven by a simple decision rule of who sits next to whom, but in body contact. From an evolutionary standpoint, cooperating with a friend may provide high returns by decreasing the risks of exploitation and cheating and can enhance fitness benefits [33–35,98]. Even though the formation and maintenance of friendships may incur certain costs, the corresponding benefits are expected to outweigh them, resulting in an overall advantageous outcome for friendship to be selected as a viable strategy [78,98]. Interestingly, no substantial impact of social tolerance favouring cooperation was found. There are two possibilities to explain this juxtaposition: first, since social tolerance is a prerequisite for forming and maintaining friendships [48,73], an underlying enhanced tolerance among friends might have obscured the effects of our co-feeding measure. Yet no correlation was found between dyadic social tolerance and friendship, although it is essential to mention that the criterion for the social tolerance phase was satisfied. The second possible explanation is the current methodology's limitation in assessing tolerance among dyads that were never together at the training apparatus during the co-feeding assay, thus partly creating an all-or-nothing variable. Besides, the current measure of tolerance was taken during an initial training phase, and task characteristics could influence the individuals’ participation, too (but see [73]). We recognize the need for a more robust co-feeding tolerance measure in future investigations. For instance, adopting a ‘co-feeding plot’ approach could provide comprehensive information at both group as well as dyad levels [99].

Homophily in social personality traits—in this case, affiliation—exhibited no discernible impact on cooperation. Since we did not find any collinearity of affiliation with social tolerance or friendship, this might imply that the observed affiliation trait scores were contingent on partner-specific affiliative interactions. It would be worthwhile to investigate whether this selective yet discriminative mechanism is exclusive to despotic societies due to interdependencies (see [69]). Therefore, in socially tolerant species, homophily in social personality traits, in general, can be expected to act as a stronger and more efficient force driving cooperation. An alternative explanation would be the strong effect of friendship masking any potential influence of homophily in affiliation on cooperation. Compared to some previous studies (see [48,100]), we did not find friendships to be driven by personality homophily. This could be attributed to the use of only one proxy, contact sit, and not grooming to define friendship. While combining the two cannot be achieved in this study due to data dependence, i.e. grooming was a variable in our personality assessment, future studies should be designed to collect independent measures of these variables to understand the evolutionary origins of homophily better. Nevertheless, we investigated convergent validity by looking at the correlation between dyadic contact sit and grooming and provided evidence that they are highly positively correlated. Finally, we recommend disentangling the effect of individual personalities from dyad-specific measures on cooperation in future studies [101].

In contrast to personality homophily, we found personality heterophily as a proximate mechanism fostering cooperation. Less-explorative and -active individuals were likely to form cooperative dyads with more-explorative and -active ones. These findings are in line with our prediction that heterophily in non-social personality traits can positively influence cooperation, potentially due to varying problem-solving perspectives of the associated individuals. From a relatively simple cognitive point of view, dyads with non-social personality differences may represent a ‘leader–follower’ relationship ([102], also see [103]). To illustrate, partners with greater explorative tendencies or higher activity levels may function as ‘catalysts’ (cf. [57]), while their counterparts with lower explorative behaviour or reduced activities may follow them. This is in contrast to dyads with either two ‘leaders’ or two ‘followers’, which may have problems coordinating in a cooperation set-up, such as the one tested in this study. From an evolutionary viewpoint, such a relationship can enable leaders to gain by imposing their preferences on followers but simultaneously reducing conflicting preferences and the ‘cost of consensus’ [103].

## 5. Conclusion

In summary, we illustrated that friendships can strongly predict cooperative dyad formation and its success. Lower disparity in dominance ranks can lead to higher cooperative task success. We also elucidated the potential reasons behind the limited predictive power of kinship and social tolerance concerning cooperation. Finally, we tested the hypothesis that personality homophily and heterophily may act independently at the trait level to drive cooperation. Yet, we only found support for heterophily in the non-social traits linked to cooperation. Additionally, we highlighted a few shortcomings of our study—the lack of a delayed version of the cooperation task (control) to better explain the macaques' understanding of the need for partners, the inability to assess friendship using a composite measure of proximity, grooming and contact sit (but see [48]), and the lack of a robust social tolerance measure (but see [73]). Nevertheless, we provided a comprehensive account of three broad proximate mechanisms (social relationships, social status and personalities) of cooperation and its decision rules (for partner choice) in a group-living primate species. We specifically emphasized the low cognitive demands of these mechanisms yet highlighted their broad evolutionary implications for a better understanding of the evolution of cooperation.

## Data Availability

Data and codes have been deposited in the Open Science Framework and can be accessed using the link https://doi.org/10.17605/OSF.IO/HCG8A [104]. Supplementary material is available online [105].
